# Odontological Society in London

**Published:** 1857-04

**Authors:** 


*Odontological Society in London.*—In the last number of the Journal we took occasion to state that a meeting had been held in London by a number of dentists of that metropolis, preliminary to the permanent organization of a society. It will be seen by the following, which we copy from the Medical Times & Gazette of the 10th of January, that such organization has taken place.—Eds.

"The first meeting of this society was held on Monday evening last, when a large number of the leading metropolitan and provincial practitioners of dental surgery met together. Some preliminary business having been gone through, the president, Mr. Cartwright, delivered an able and eloquent address, in which, after glancing at the progress of dental surgery during the last century, he took a review of the present position and prospects of the profession, and strongly urged the necessity of a liberal education in conjunction with the special qualification required for those who would practice this department of surgery with credit and success. The advisability of maintaining the connection of dental with general surgery was strongly insisted upon, and it was held that a *voluntary* separation from the college of surgeons could not but be disadvantageous to the body of dentists. The great need of a society formed on the model of other scientific societies, as a point of union among the practitioners of dental surgery and as a medium for the communication of experience and the discussion of professional subjects, was pointed out, and the president concluded by expressing his conviction that these objects would be fully attained by the establishment of the Odontological Society. He then urged upon the members the necessity of cordial co-operation in furthering the purposes of the society by the contribution of papers of interest, and by attendance at the meetings. Several interesting preparations illustrative of dental pathology were exhibited, and there was a good display of instruments and appliances interesting to the profession. After the meeting the gentlemen present and many friends of the society partook of an elegant supper at the house of Mr. Saunders, the treasurer. The great unanimity which characterised the proceedings of the evening gave earnest of the vigor with which the objects of the society will be carried out."

